# Antibiotic Resistance Profiles of Commensal and Pathogenic Bacteria Isolated from Wild Boar Carcasses in Campania Region, Southern Italy

**DOI:** 10.3390/antibiotics15010065

**Published:** 2026-01-07

**Authors:** Claire Julie Akwongo, Kurt Houf, Lorena Sollena, Luca Borrelli, Alessandro Fioretti, Nicoletta Murru, Maria Francesca Peruzy

**Affiliations:** 1 Department of Veterinary Medicine and Animal Production, University of Naples Federico II, Via Federico Delpino 1, 80137 Naples, Italy; clairejulie.akwongo@unina.it (C.J.A.); lorenasollena99@gmail.com (L.S.); luca.borrelli@unina.it (L.B.); fioretti@unina.it (A.F.); mariafrancesca.peruzy@unina.it (M.F.P.); 2 Department of Veterinary and Biosciences, Faculty of Veterinary Medicine, Ghent University, Salisburylaan 133, 9820 Merelbeke, Belgium; kurt.houf@ugent.be; 3 Task Force on Microbiome Studies, University of Naples Federico II, 80137 Naples, Italy

**Keywords:** antibiotic resistance, One Health, epidemiology, wild boar

## Abstract

**Background/Objectives**: Antimicrobial resistance (AMR) in wildlife is an emerging public health concern due to the risk of zoonotic transmission, especially through the food chain, yet data on free-ranging animals remain scarce. This study examined the presence and patterns of AMR among bacteria isolated from hunted wild boars in the Campania region of Italy. **Methods**: Matrix-assisted laser desorption ionization time of flight mass spectrometry (MALDI-TOF MS) was used to identify bacterial isolates from wild boar meat and carcass swabs to the species level, and the Kirby–Bauer disk diffusion test was applied to screen 205 isolates, spanning 20 bacterial genera, against a panel of clinically relevant antibiotics. Resistance metrics were analyzed at genus and antibiotic levels, and patterns were visualized using a hierarchically clustered heatmap. **Results**: Resistance was detected in 15 of the 20 genera, with full susceptibility observed in *Acinetobacter*, *Arthrobacter*, *Glutamicibacter*, *Leclercia*, and *Rahnella*. Overall, 67.3% (138/205) of the isolates showed resistance to at least one antibiotic, with 33.7% (69/205) classified as multidrug-resistant (MDR). Carbapenems retained the highest activity (≥95% susceptibility) among all genera tested, while amoxicillin/clavulanate (78.4%) and aztreonam (57.4%) exhibited the highest mean resistance. Among potential pathogens, *Escherichia coli* exhibited an extended-spectrum β-lactamase (ESBL)-like phenotype, with resistance to amoxicillin/clavulanate (67%), aztreonam (54%), and ceftazidime (47%) but preserved carbapenem susceptibility. *Staphylococcus* spp. showed pronounced resistance to linezolid (57%) and erythromycin (52%), whereas *Pseudomonas* isolates demonstrated elevated resistance to aztreonam and ceftazidime (57% each). Opportunistic pathogens such as *Alcaligenes faecalis* and *Pantoea agglomerans* showed peak resistance to ciprofloxacin and amoxicillin/clavulanate. Pathogens and opportunistic pathogens demonstrated higher mean resistance (>30%) than commensals (≤32%), but the difference in mean and median resistance levels was not statistically significant (Mann–Whitney’s U test, W = 4, *p* = 0.39). **Conclusions**: These findings highlight the widespread occurrence of AMR and MDR phenotypes, with clinically significant resistance patterns in wild-boar-associated bacteria, including non-pathogenic strains, highlighting their role in the amplification of AMR. Although the preservation of carbapenem susceptibility underscores their potential as last-line antibiotics, the high resistance to commonly used antibiotics raises concerns for zoonotic transmission. Surveillance of wildlife reservoirs therefore remains critical for integrated AMR control.

## 1. Introduction

Antimicrobial resistance (AMR) is a natural evolutionary process in which microorganisms, including bacteria, viruses, and fungi, develop the ability to adapt to and resist the effects of drugs that once killed or inhibited them, rendering them ineffective [1]. In recent years, there has been a global surge in AMR and the emergence of multidrug-resistant bacteria (MDR), posing a public health threat [2]. This has primarily been attributed to the overuse and misuse of antimicrobial agents in both human and veterinary medicine, as well as in agriculture [3].

While society grapples with the consequences of misuse and overuse of antimicrobials, emerging sources of AMR have become a focal point of scientific research. One such area of interest is the potential role of wildlife in the spread of AMR. Studies have shown that wild animals can harbor a variety of bacteria, including strains carrying multiple resistance genes that can be exchanged between pathogenic and non-pathogenic organisms [4,5]. Subsequently, these can be transferred to humans during the handling, processing, and consumption of wild game meat, creating a direct pathway for the spread of antimicrobial resistance [6]. Furthermore, increased interaction between wildlife, domestic animals, and humans provides an interface for horizontal gene transfer, especially in environments contaminated with antimicrobial residues or resistant microbes. Such interactions facilitate the dissemination of resistance determinants across species and ecosystems, amplifying the AMR risk [7,8].

While much focus has been placed on pathogenic bacteria as the primary drivers of AMR transmission, non-pathogenic bacteria, often referred to as commensal or environmental bacteria, play an equally critical but largely unexplored role. These bacteria, which naturally inhabit the gut, skin, and mucosal surfaces of animals and humans, as well as soil and water environments, frequently harbor resistance genes within their genomes or in mobile genetic elements such as plasmids, transposons, and integrons [9]. Although these bacteria may not directly cause disease, they act as silent reservoirs of resistance genes that can be transferred to pathogenic species through horizontal gene transfer mechanisms like conjugation, transformation, and transduction. This gene exchange potential therefore enables non-pathogenic bacteria to become essential players in the environmental resistome [10,11].

In food production systems, commensal bacteria such as *Escherichia coli* and *Enterococcus* spp. and *Lactobacillus* spp. can acquire and disseminate resistance determinants within the microbiota of animals [12]. These bacteria persist throughout slaughtering, meat processing, and handling stages, contaminating carcasses and the environment, especially in less ideal hygienic conditions [13]. Once in the food, they can colonize the human gut, where they may exchange resistance genes with opportunistic or pathogenic bacteria, thus forming an invisible bridge between environmental and clinical reservoirs of AMR [11,14]. In wild animals, such bacteria may be naturally exposed to resistant strains from agricultural runoff, contaminated water, or contact with livestock, allowing them to accumulate and maintain resistance genes even in the absence of antibiotic exposure [15].

Wild boars are particularly relevant in this issue. As omnivorous animals with scavenging and rooting behaviors, they frequently encounter environments contaminated with antimicrobial residues, fecal bacteria, and resistant organisms from livestock and human waste [16]. This exposure facilitates colonization by resistant bacteria, many of which may persist in their gut and skin flora. Given their popularity as game animals, wild boars are hunted for both sport and culinary purposes in various regions across the globe, and their meat has become an integral part of local diets, contributing to the economic and cultural fabric of communities [17]. However, the very practices that sustain this relationship between humans and wild boars may also foster a conducive environment for the spread of resistant bacteria, given their role as potential carriers of resistant bacteria, both through direct contact and the consumption of contaminated meat. Considering the potential for the spread of AMR through the food chain, efforts to combat antimicrobial resistance should therefore not be limited to healthcare settings but must extend to include comprehensive surveillance and regulation within the food production and distribution systems [18].

While several studies have reported AMR in wild boar populations in Italy [19,20] and other parts of the world [21], they have primarily focused on pathogenic bacteria recovered from fecal samples. Consequently, a critical knowledge gap persists regarding the resistance profile of bacteria present on carcasses and in edible meat, which is directly relevant to public health. Moreover, globally, studies have focused on screening pathogenic bacteria but have largely neglected non-pathogenic and environmental strains. Such bacteria may not pose an immediate health threat but may serve as important intermediaries for resistance gene transfer during food handling or within the human gut following ingestion [22]. As a result, neglecting non-pathogenic bacteria in surveillance programs underestimates the overall burden and complexity of AMR transmission in food systems.

Comprehensive epidemiological studies that incorporate both pathogenic and commensal bacteria from wild boar populations, as well as in the meat destined for human consumption, are essential for understanding the scope of the problem, as well as providing a comprehensive understanding of the dynamics of AMR transmission, including the potential for spillover to human and domestic animal populations [16]. Considering this, the present study aims to unravel the AMR profiles of commensal and pathogenic bacteria isolated from wild boar carcasses. Specifically, we seek to determine the potential role of wild boar meat in the transmission of AMR to humans. We aim to emphasize the critical need to expand monitoring programs to include non-pathogenic bacteria as key indicators of resistance dissemination, thereby providing the basis for informed mitigation strategies and promoting food safety.

## 2. Materials and Methods

### 2.1. Sourcing and Identification of Isolates

The study assessed antibiotic the susceptibility of 205 bacterial isolates from the strain collection from a previous study by Peruzy et al. [23], which isolated bacteria from carcass swabs and meat of 36 wild boars hunted between October and December 2019 from the Campania region of Italy. The isolates were stored at −20 °C at the microbiology laboratory at the Department of Veterinary Medicine and Animal Production, University of Naples Federico II. Pure colonies of all isolates were re-identified by MALDI TOF-MS, as specified by the manufacturer (Bruker Daltonics, Bremen, Germany). Spectral acquisition and analysis were performed using the MALDI BioTyper’s, MBT Compass^®^ HT (Bruker Daltonics, Bremen, Germany). A score of ≥2 was considered valid for species-level identification, while that between 1.7 and 2 was considered valid for genus-level identification. A score ≤ 1.7 was considered invalid.

### 2.2. Antibiotic Susceptibility Testing

**Figure 1:**
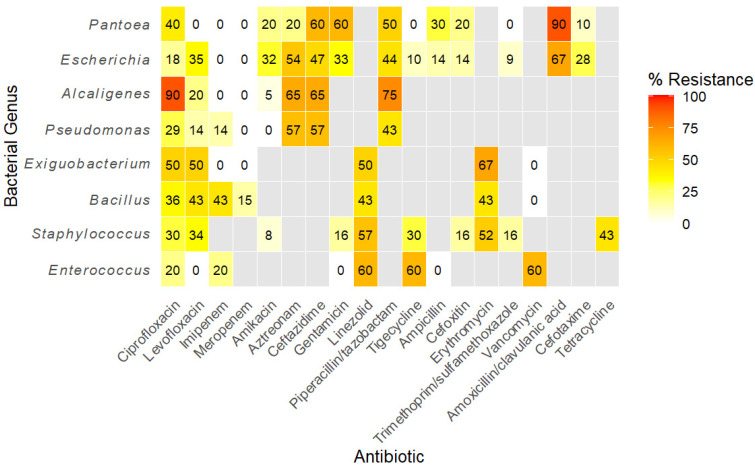
Heatmap showing the distribution of antibiotic resistance among bacterial genera based on isolate-level testing. Resistance values are expressed as the percentage of isolates resistant to each antibiotic. Genera tested against similar antibiotics are clustered together. Grey cells denote combinations for which testing was not performed.

The Kirby–Bauer disk diffusion method for screening for antibiotic resistance was performed according to the European Committee on Antimicrobial Susceptibility Testing [24] (EUCAST) protocol using a panel of commonly used antibiotics (antibiotic disks ROSCO NEO-SENSITABS^TM^; sourced from Rosco Diagnostica A/S Taastrugaardsvej 30, DK-2630, Taastrup, Denmark). The choice of antibiotics tested for each organism was based on whether they were indicated for use against the specific bacteria and whether EUCAST clinical breakpoints for the antibiotics were available. The antibiotics disks included were ampicillin (10 µg), ampicillin (2 µg), amoxicillin–clavulanate (3 µg), aztreonam (30 µg), benzyl penicillin (1 U), ceftazidime (10 µg), cefoxitin (30 µg), cefotaxime (5 µg), erythromycin (15 µg), gentamycin (30 µg), imipenem (10 µg), levofloxacin (5 µg), linezolid (10 µg), meropenem (10 µg), oxytetracycline (30 µg), piperacillin–tazobactam (36 µg), tetracycline (10 µg), tigecycline (15 µg), trimethoprim–sulphamethoxazole (25 µg), and vancomycin (5 µg), as indicated in Figure 1 and Supplementary Material Table S1. A fresh culture of the bacteria was diluted in a normal saline solution, and its turbidity was adjusted to 0.5 McFarland’s standard before inoculating a loopful onto a Mueller–Hinton agar plate (OXOID, Basingstoke, Hampshire, United Kingdom; catalog number CM0131B) (for non-fastidious) or Mueller–Hinton agar + 5% defibrinated horse blood and 20 mg/L β-NAD (OXOID, Basingstoke, Hampshire, United Kingdom; catalog number PB1229A) (for fastidious organisms). The antibiotic disks were placed onto the inoculated agar plates and incubated at 37 °C for 18–24 h for non-fastidious organisms or in the presence of CO_2_ at 37 °C for 18–24 h for fastidious organisms. The inhibition zone was measured, and readings were compared with those specified in the EUCAST clinical breakpoint table [25] to categorize the organism as susceptible or resistant. For organisms for which no breakpoints were available, comparisons were made based on clinical breakpoints for other organisms closely related to them (Table 1). Organisms were classified as MDR if they showed resistance to ≥3 antibiotic classes in accordance with the classification criterion by [26].

### 2.3. Statistical Analysis

Statistical analysis was performed in the CRAN project’s R statistical software version 2025.05.1 + 513 [27]. Descriptive statistics were used to describe the main characteristics of the antimicrobial resistance profiles of the bacterial isolates from wild boar carcasses. Frequencies and percentages were calculated to summarize categorical variables, and median, means, and standard deviations were used to summarize numerical variables. Heatmaps of resistance to specific antibiotics were constructed using the R package ggplot2 for genera of bacteria with at least 5 isolates tested. Differences in resistance levels between pathogens and non-pathogenic bacteria were assessed by the Mann–Whitney U test as described in [28]. *p*-values ≤ 0.05 were considered significant.

## 3. Results

### 3.1. Bacterial Isolates Tested

The 205 isolates studied included both Gram-positive and Gram-negative bacteria, some of which are considered pathogenic, while others are commensals or environmental, belonging to 20 bacterial genera, dominated by *Staphylococcus*, *Escherichia*, *Alcaligenes*, and *Bacillus*, as shown in Table 2.

### 3.2. Antibiotic Resistance Profiles

As shown in Table 3, resistance to antibiotics was detected among bacteria belonging to 15 of the 20 genera (75%), while 5 (20%) including *Acinetobacter*, *Fictibacillus*, *Glutamicibacter*, *Leclercia*, and *Rahnella* were fully susceptible. Overall, 138 of the 205 isolates (67.3%) were resistant to at least one antibiotic, of which 7 were resistant to only one antibiotic, 11 to two, and 120 to three or more antibiotics. Additionally, 69 isolates (33.7%) were classified as multidrug-resistant.

### 3.3. Antibiotic Resistance Patterns

Antibiotic resistance patterns were analyzed for eight genera of bacteria that had representative isolates greater than five. The overall median resistance was 35% (IQR 14–58%). Susceptibility to carbapenems (imipenem and meropenem) was retained across all genera (≥95%). Conversely, the highest mean resistance was recorded for amoxicillin/clavulanate and aztreonam (78.4% and 57.4%, respectively) (Table 4).

A hierarchically clustered heatmap (Figure 1) partitioned isolates into two principal groups: Gram-negative genera characterized by extensive β-lactam and aztreonam resistance and Gram-positive genera distinguished by resistance to protein synthesis inhibitors (tigecycline, linezolid, and tetracycline). Genus-specific profiles revealed that the opportunistic pathogens *Alcaligenes* and *Pantoea* showed the highest single-drug resistance (ciprofloxacin and amoxicillin/clavulanate, each 90%). Among primary pathogens, *Escherichia* displayed a classical extended-spectrum β-lactamase phenotype with high resistance to amoxicillin/clavulanate (67%), aztreonam (54%), and ceftazidime (47%) but full susceptibility to carbapenems. *Pseudomonas* showed elevations in aztreonam and ceftazidime resistance (57% each). *Staphylococcus* demonstrated pronounced linezolid (57%) and erythromycin (52%) resistance, while *Enterococcus* and *Exiguobacterium* shared similarly elevated tigecycline and linezolid resistance (≤67%).

As shown in Table 5, true pathogens and opportunistic pathogens presented higher mean resistance (>30%) and extreme peaks (>90%) than commensal/environmental genera (mean ≤32%, peaks ≤67%), but carbapenem activity (<15%) was maintained across pathogenic categories. However, the levels of resistance to antibiotics did not differ significantly between the groups (Mann–Whitney’s U test, W = 4, *p* = 0.39).

## 4. Discussion

The phenotypic antibiotic susceptibility testing of 205 bacterial isolates spanning 20 genera, obtained from wild boar carcass swabs and meat samples in the Campania region of Italy, reveals a complex landscape of resistance that mirrors both wildlife ecology and anthropogenic pressures.

Overall, 67.3% (138/205) of the isolates exhibited resistance to at least one antibiotic, and 33.7% (69/205) were MDR, highlighting the substantial carriage of resistant bacteria in free-ranging wild boars. Other Italian studies likewise reported MDR prevalences in bacteria isolated from wild boars ranging from 5.6% to 100%. These included 5.6% among *Salmonella* spp. isolates from spleens, livers, and rectal swabs of wild boars from Tuscany [20], 62.5% among nasal swab *Enterococcus* spp. and *Staphylococcus* spp. isolates from Campania [64], and 100% among *Escherichia coli* from mesenteric lymph nodes and feces of 23.3% of wild boars sampled in northern Italy [65]. These studies suggest a consistent reservoir function of wild boars across regions. While the figures in this study are comparable to other Italian studies, there is a consistent trend of higher prevalences being reported in the south than in the north of Italy. The elevated resistance in this study could reflect local AMR selection pressures either from anthropogenic environmental contamination or agricultural antibiotic use, as well as regional ecological factors, as reported by Karwowska [66]. Notably, intensive farming activity is prevalent across the Campania region, potentially promoting environmental dissemination of resistant bacteria through run-offs, manure application, or water systems [67]. The MDR prevalences in this study are also comparable to those reported in other European studies; for example, a study by Sabença et al. [68] in Portugal identified 44% MDR prevalence among *E. coli* isolates from wild boars, while a German study by Günther et al. [69] reported a 56% prevalence of MDR among *E. coli*.

The public health implications of these findings are profound. Due to their rapidly expanding populations and intensified contact with agricultural and peri-urban landscapes, wild boars represent a significant ecological interface for the transmission of resistant bacteria [16]. The carriage of MDR bacteria in these animals introduces the possibility of gene flow between environmental, animal, and huma microbiota, especially through hunting or food contamination [70,71].

While MDR organisms are a big concern, ESBL and carbapenemase producers are particularly urgent global health threats, with related infections associated with mortality rates of up to 50%. Moreover, the development of resistance to these often last-resort antibiotics leads to the dependence on older, more toxic drugs such as colistin, presenting a further challenge to health management [72]. In this study, Gram-negative genera demonstrated resistance predominantly to β-lactams and monobactams. Particularly, resistance to amoxicillin/clavulanate (67%), aztreonam (54%), and ceftazidime (47%) alongside universal susceptibility to imipenem and meropenem (carbapenems) was observed among *Escherichia* isolates, consistent with the ESBL phenotype as described by Husna et al. [73]. This pattern mirrors findings from other wild boar studies in Europe, where resistance to carbapenems remains low; 0% as reported by Rega et al. [74], Selmi et al. [75], and Pătrînjan et al. [76], and 5.9% by Holtmann et al. [77], while extended-spectrum cephalosporins show high resistance frequencies (up to 85%) [75,76,78]. The preservation of carbapenem activity suggests limited selective pressure for carbapenemase genes in the wild.

Among Gram-positive genera (including *Staphylococcus* spp. and *Enterococcus* spp.), resistance to protein synthesis inhibitors, including linezolid, tigecycline, and erythromycin, was dominant. Macrolide (erythromycin) resistance is commonly detected in wild-boar-associated bacterial isolates, while tigecycline resistance remains principally a clinical and production-animal concern and is rarely reported in primary wild boar field studies [64,79]. For example, Poeta et al. [80] found 48.5% erythromycin resistance among fecal *Enterococcus* isolates from Portuguese wild boars, while a meta-analysis by Akwongo et al. [81] reported a 22% macrolide resistance across studies. Studies by [82] reported phenotypic resistance to linezolid as well as the carriage of the transferable genes optrA and poxtA that are responsible for linezolid resistance in *E. faecalis* and *E. feacium* isolates, from in wild boars in Italy. Additionally, linezolid resistance in *Enterococcus* spp. isolates in other Italian wildlife was also reported by Smoglica et al. [83], albeit at low levels. While resistance to linezolid remains rare in wildlife [81,82,83], there have been more frequent reports in livestock-associated *S. aureus* [84,85,86] and *Enterococcus* spp. [87,88], suggesting possible cross-species transmission routes [88,89,90]. Linezolid resistance in wild boars and other wildlife, especially those that are hunted for human consumption, is particularly concerning and warrants close surveillance to prevent its transmission through the food chain, as it is considered a last-resort antibiotic for MDR Gram-positive infections [91].

Opportunistic pathogens such as *Alcaligenes faecalis* and *Pantoea agglomerans* exhibited the highest single-drug resistance peaks (90%) to ciprofloxacin and amoxicillin/clavulanate, indicating their ability to accumulate resistance determinants even in wildlife hosts. Previous studies have reported extensively resistant (XDR) and pan-drug-resistant (PDR) *Alcaligenes faecalis*, with resistance rates as high as 83.6% in human studies [31,92]. Moreover, recent studies have also identified high-level quinolone resistance in environmental isolates of *Alcaligenes faecalis*, attributing it to efflux pump overexpression and chromosomal mutations [93,94]. By contrast, some environmental commensals such as *Rahnella aquatilis* remained fully susceptible. This dichotomy echoes European surveillance data, which often demonstrates elevated quinolone and β-lactam resistance in opportunistic Gram-negatives from wildlife, likely driven by agricultural run-offs and contaminated water and feed sources, while purely environmental bacteria genera retain susceptibility [95,96]. The complete susceptibility of some of the environmental genera may also suggest variability in environmental exposure and intrinsic resistance mechanisms.

Furthermore, true pathogens and opportunistic pathogens showed higher resistance metrics than commensals or environmental isolates. True pathogens and opportunistic pathogens demonstrated mean resistance rates exceeding 30%, with peaks reaching 90%, in contrast to the generally lower rates observed in environmental isolates. This association has been documented elsewhere, likely due to increased selective pressures faced by pathogenic bacteria either in hosts previously exposed to antibiotics or through horizontal gene transfer in microbiomes with higher antibiotic exposure [6,97]. This trend is also supported by the One Health framework, which posits that zoonotic pathogens, due to frequent host transitions and environmental adaptability, often acquire and maintain resistance genes [98]. Notably, *S. aureus* and *E. coli*, both well characterized pathogens, exhibited classical resistance profiles, including linezolid and β-lactam resistance, respectively, patterns that have frequently been documented in clinical and environmental studies [99,100]. Despite the higher resistance metrics observed among pathogens than environmental bacteria, the difference is not statistically significant. This therefore reinforces the argument that non-pathogenic bacteria can equally contribute to amplification of AMR along the food production chain, as suggested in [22].

## 5. Conclusions

The findings of this study provide compelling evidence that wild boars are significant reservoirs of antibiotic-resistant bacteria, including MDR strains of species with clinical relevance. The observed resistance patterns reflect both bacterial phylogeny and pathogenic status, with notable differences between Gram-positive and Gram-negative strains. Despite the extensive resistance profiles observed, susceptibility to carbapenems and amikacin was generally conserved across all genera, which emphasizes the critical importance of these antibiotics as last-resort treatments and highlights the need for stringent control of their use in both human and veterinary medicine contexts. However, the presence of resistance to other potent antibiotics, even if at low levels, raises concerns over potential future shifts in resistance if selection pressures increase.

Further, the resistance patterns observed in Campania suggest a more diverse and possibly advanced development of resistance across both environmental and clinically significant bacteria. This trend may reflect ecological pressures within the Mediterranean biome, driven by high human–wildlife interaction and dense agricultural land use, as well as variability in resistance mechanisms such as intrinsic resistance among bacteria. Furthermore, the presence of resistance in bacteria like *Macrococcus* spp. and *Rothia* spp., organisms often dismissed as harmless commensals, even at low percentages, supports emerging views that these non-pathogenic organisms can act as reservoirs or conduits for resistance genes, potentially transferring them to more virulent or invasive species.

While this study provides relevant information regarding antibiotic resistance profiles of pathogenic and non-pathogenic bacterial isolates from wild boar carcasses, it is limited to phenotypic resistance. A further investigation to unravel the resistance profiles and patterns at the genotypic level is recommended to provide a clearer understanding of the underlying resistance mechanisms.

## Figures and Tables

**Table 1:** EUCAST clinical breakpoints applied to interpret antimicrobial susceptibility test results of the different genera of bacteria.

Genera	Breakpoint Available		Breakpoint Used	Quality Control Strains	Reason for Choice
	Yes	No			
*Acinetobacter*	✓		*Acinetobacter*	*Pseudomonas aeruginosa* ATCC 27853	-
*Alcaligenes*		✓	*Pseudomonas*	*Pseudomonas aeruginosa* ATCC 27853	Taxonomically related to *Pseudomonas*
*Arthrobacter*		✓	*Corynebacterium*	*Staphylococcus aureus* ATCC 29213	Taxonomically related to *Corynebacterium* (Phylum Actinobacteria)
*Bacillus*	✓		*Bacillus*	*Staphylococcus aureus* ATCC 29213	-
*Enterobacter*	✓		*Enterobacteriaceae*	*Escherichia coli* ATCC 25922	-
*Enterococcus*	✓		*Enterococcus*	*Enterococcus faecalis* ATCC 29212	-
*Escherichia*	✓		*Enterobacteriaceae*	*Escherichia coli* ATCC 25922	-
*Exiguobacterium*		✓	*Bacillus*	*Staphylococcus aureus* ATCC 29213	Taxonomically related to *Bacillus*
*Fictibacillus*		✓	*Bacillus*	*Staphylococcus aureus* ATCC 29213	Reclassified from *Bacillus*
*Glutamicibacter*		✓	*Corynebacterium*	*Staphylococcus aureus* ATCC 29213	Taxonomically related to *Corynebacterium* (Phylum Actinobacteria)
*Kocuria*		✓	*Staphylococcus*	*Staphylococcus aureus* ATCC 29213	Closely related to *Staphylococcus* (family Micrococcaceae)
*Leclercia*	✓		*Enterobacteriaceae*	*Escherichia coli* ATCC 25922	-
*Macrococcus*		✓	*Staphylococcus*	*Staphylococcus aureus* ATCC 29213	Closely related to *Staphylococcus* (family Micrococcaceae)
*Paenarthrobacter*		✓	*Corynebacterium*	*Staphylococcus aureus* ATCC 29213	Taxonomically related to *Corynebacterium* (Phylum Actinobacteria)
*Pantoea*	✓		*Enterobacteriaceae*	*Escherichia coli* ATCC 25922	-
*Pseudomonas*	✓		*Pseudomonas*	*Pseudomonas aeruginosa* ATCC 27853	-
*Rahnella*	✓		*Enterobacteriaceae*	*Escherichia coli* ATCC 25922	-
*Rothia*		✓	*Staphylococcus*	*Staphylococcus aureus* ATCC 29213	Reclassified from *Staphylococcus*
*Staphylococcus*	✓		*Staphylococcus*	*Staphylococcus aureus* ATCC 29213	-
*Streptococcus*	✓		*Streptococcus* group A, B, C, G	*Streptococcus pneumoniae* ATCC 49619	-

**Table 2:** Composition and description of bacterial isolates for which antimicrobial susceptibility tests were performed. Isolates were recovered from hunted wild boars from the Campania region, Italy.

Genus	Species	Gram-Stain	Pathogenicity	Comments	Number of Isolates	Reference
*Acinetobacter*	*Acinetobacter lwoffii*	Gram-negative	Opportunistic pathogen	Gram-negative aerobic bacilli, normal flora of skin, oropharynx, and perineum. Can cause disease in immunocompromised individuals.	1	[29,30]
*Alcaligenes*	*Alcaligenes faecalis*	Gram-negative	Opportunistic pathogen	Infections are often difficult to treat due to increased resistance, with MDR and XDR patterns.	20	[31]
*Arthrobacter*	*Arthrobacter koreensis*	Gram-positive	Non-pathogenic	Soil bacteria, *Arthrobacter koreensis* is non-pathogenic, though other species of the same genera such as *Arthrobacter woluwensis* have been reported to cause disease in humans.	1	[32,33]
*Bacillus*	*Bacillus cereus* (5), *Bacillus megaterium* (1), *Bacillus mycoides* (3), *Bacillus pumilus* (1), *Bacillus simplex* (3), *Bacillus subtilis* (1)	Gram-positive	Opportunistic/pathogenic	Some species, e.g., *B. cereus*, are pathogens, while some are not. Certain species, e.g., *B. subtilis* and *B. pumilus*, are used as probiotics due to their ability to produce antimicrobial compounds.	14	[34,35]
*Enterobacter*	*Enterobacter ludwigii*	Gram-negative	Opportunistic pathogen	Common in healthcare-associated infections, XDR infections have been reported.	1	[36,37]
*Enterococcus*	*Enterococcus casseliflavus* (1), *Enterococcus faecalis* (1), *Enterococcus mundtii* (1)	Gram-positive	Gut commensal and Opportunistic pathogen	Major nosocomial pathogen. *E. faecalis* is one of the most common causes of MDR hospital infections.	5	[38]
*Escherichia*	*Escherichia coli* (56), *Escherichia marmotae* (1)	Gram-negative	Gut commensal/pathogenic	*E. coli* is A normal flora, but some strains, e.g., *Escherichia coli* O157:H7, are important pathogens.	57	[39,40]
*Exiguobacterium*	*Exiguobacterium mexicanum*	Gram-positive	Mostly commensal	Rarely implicated in infection.	6	[41]
*Fictibacillus*	*Fictibacillus arsenicus*	Gram-positive	Non-pathogenic	Not pathogenic, used as a nematicide.	1	[42]
*Glutamicibacter*	*Glutamicibacter arilaitensis*	Gram-positive	Non-pathogenic	Formerly belonged to *Arthrobacter* genus. Commonly found on cheese rinds, sometimes used in starter cultures and as A plant-growth-promoting bioinoculant.	1	[43,44]
*Kocuria*	*Kocuria rhizophila*	Gram-positive	Mostly commensal	Rare opportunistic infections, mostly device-associated and in immunocompromised individuals.	4	[45,46]
*Leclercia*	*Leclercia adecarboxylata*	Gram-negative	Opportunistic pathogen	Rarely isolated from clinical samples, but infections reported in immunocompromised individuals and polymicrobial infections in immunocompetent patients, mostly susceptible to antibiotic treatment.	1	[47,48]
*Macrococcus*	*Macrococcus canis*	Gram-positive	Commensal/opportunistic pathogen	Closely related to *Staphylococcus.* Isolates have been reported to carry mobilizable and transferable determinants of methicillin resistance, including mecB and mecD.	2	[49,50]
*Paenarthrobacter*	*Paenarthrobacter ilicis*	Gram-positive	Non-pathogenic	Soil-associated bacteria reclassified from *Arthrobacter.*	1	[51,52]
*Pantoea*	*Pantoea agglomerans*	Gram-negative	Opportunistic pathogen	Plant-associated bacteria. Can cause disease in the immunocompromised.	10	[53,54]
*Pseudomonas*	*Pseudomonas fluorescens* (2), *Pseudomonas fragi* (3), *Pseudomonas lundensis* (2)	Gram-negative	Opportunistic pathogen	*P. aeruginosa* is a key nosocomial pathogen.	7	[55]
*Rahnella*	*Rahnella aquatilis*	Gram-negative	Commensal/opportunistic pathogen	Rare infections; environmental isolate.	1	[56,57]
*Rothia*	*Rothia nasimurium*	Gram-positive	Commensal/opportunistic pathogen	Part of the normal flora of the mouth and nasopharynx. Infection with *Rothia nasimurium* has only been documented in animals, but other *Rothia* species cause infection in humans; associated with endocarditis and periodontitis.	2	[58,59,60]
*Staphylococcus*	*Staphylococcus aureus* (3), *Staphylococcus capitis* (7), *Staphylococcus chromogenes* (5), *Staphylococcus epidermidis* (2), *Staphylococcus equorum* (2), *Staphylococcus haemolyticus* (4), *Staphylococcus hominis* (1), *Staphylococcus hyicus* (14), *Staphylococcus pasteuri* (1), *Staphylococcus saprophyticus* (2), *Staphylococcus simulans* (22), *Staphylococcus warneri* (5)	Gram-positive	Skin commensal/pathogenic	Includes species like *S. aureus*, which is a major pathogen, and *S. epidermidis*, which is commensal.	67	[61,62]
*Streptococcus*	*Streptococcus gallolyticus* (1), *Streptococcus porcinus* (1), *Streptococcus suis* (1)	Gram-positive	Commensal/pathogenic	Many species are normal flora of the skin, throat, and mouth, but some are opportunistic pathogens causing mild-to-severe infections.	3	[63]
Total					205	

**Table 3:** Antimicrobial resistance profiles of 205 bacteria isolated from wild boars from Campania region, Italy.

Genera	Number Tested	Resistant (%)	MDR (%)	Susceptible (%)	Resistant Species
*Acinetobacter*	1	0 (0)	0 (0)	1 (100)	None
*Alcaligenes*	20	15 (75)	3 (15)	5 (25)	*Alcaligenes faecalis*
*Arthrobacter*	1	1 (100)	1(100)	0 (0)	*Arthrobacter koreensis*
*Bacillus*	14	6 (42.9)	6 (42.9)	8 (57.1)	*B. cereus* (4) *B. mycoides* (2),
*Enterobacter*	1	1 (100)	1 (100)	0 (0)	*Enterobacter ludwigii*
*Enterococcus*	5	4 (80)	2 (40)	1 (20)	*E. casseliflavus* (1) *E. mundtii* (3)
*Escherichia*	57	41 (71.9)	16 (28.1)	16 (28.1)	*E. marmotae* (1) *E. coli* (40)
*Exiguobacterium*	6	4 (66.7)	3 (0.5)	2 (33.3)	*E. mexicanum*
*Fictibacillus*	1	0 (0)	0(0)	1 (100)	None
*Glutamicibacter*	1	0 (0)	0 (0)	1 (100)	None
*Kocuria*	4	4 (100)	2 (50)	0 (0)	*K. rhizophila*
*Leclercia*	1	0 (0)	0 (0)	1 (100)	None
*Macrococcus*	2	2 (100)	0 (0)	0 (0)	*M. canis*
*Paenarthrobacter*	1	1 (100)	1(100)	0 (0)	*P. ilicis*
*Pantoea*	10	8 (80)	4 (40)	2 (20)	*P. agglomerans*
*Pseudomonas*	7	4 (57.1)	1 (14.3)	3 (42.9)	*P. fragi* (2) *P. lundensis* (1) *P. fluorescence* (1)
*Rahnella*	1	0 (0)	0(0)	1(100)	None
*Rothia*	2	1 (50)	1 (50)	1 (50)	*R. nasimurium*
*Staphylococcus*	67	45 (67.2)	27 (40.3)	22 (32.8)	*S. simulans* (15) *S. hyicus* (8) *S. capitis* (6) *S. warneri* (5) *S. aureus* (2) *S. epidermidis* (2) *S. haemolyticus* (2) *S. saprophyticus* (1) *S. chromogenes* (1) *S. pasteuri* (1) *S. equorum* (1)
*Streptococcus*	3	1 (33.3)	0 (0)	2 (66.7)	*S. gallolyticus*
Total	205	138 (67.3)	69 (33.7)	67 (32.7)	

**Table 4:** Resistance metrics for specific antibiotics.

Antibiotic	Mean Resistance (%)	Median Resistance (%)	Number of Genera Tested
Amoxicillin/clavulanic acid	78.4	78.4	2
Ceftazidime	57.4	58.6	4
Erythromycin	53.9	52.2	3
Piperacillin/tazobactam	53	47	4
Linezolid	52.4	53.4	4
Aztreonam	49.1	55.8	4
Tetracycline	43.3	43.3	1
Ciprofloxacin	39	32.8	8
Gentamicin	27.4	24.9	4
Tigecycline	25.1	20.2	4
Levofloxacin	24.6	27.1	8
Vancomycin	20	0	3
Cefotaxime	19.1	19.1	2
Cefoxitin	16.8	16.4	3
Ampicillin	14.7	14	3
Amikacin	12.8	7.5	5
Imipenem	11	0	7
Trimethoprim/sulfamethoxazole	8.4	8.8	3
Meropenem	2.5	0	6

**Table 5:** Comparison of resistance metrics stratified by pathogenic status.

Genus	Highest (%)	Antibiotic to Which There Was Highest Resistance	Lowest (%)	Antibiotic to Which There Was Lowest Resistance	Mean (%)	Status
*Alcaligenes*	90	Ciprofloxacin	0	Imipenem	40	Opportunistic pathogen
*Bacillus*	42.9	Levofloxacin	0	Vancomycin	31.7	Commensal/environmental
*Enterococcus*	60	Tigecycline	0	Ampicillin	27.5	Opportunistic pathogen
*Escherichia*	66.7	Amoxicillin/clavulanic acid	0	Imipenem	27	Pathogen
*Exiguobacterium*	66.7	Erythromycin	0	Imipenem	30.9	Commensal/environmental
*Pantoea*	90	Amoxicillin/clavulanic acid	0	Levofloxacin	26.7	Opportunistic pathogen
*Pseudomonas*	57.1	Aztreonam	0	Amikacin	26.8	Pathogen
*Staphylococcus*	56.7	Linezolid	7.5	Amikacin	30.3	Pathogen

## Data Availability

All data associated with this study have been included in the manuscript. Additional information can be obtained from the Supplementary Materials and/or by contacting the corresponding author.

## Supplementary Materials

The following supporting information can be downloaded at: https://www.mdpi.com/article/10.3390/antibiotics15010065/s1, Table S1: Resistance of different bacterial genera to different classes of antibiotics.

## Abbreviations

The following abbreviations are used in this manuscript:

AMR	Antimicrobial resistance
ESBL	Extended-spectrum beta lactamase
EUCAST	European Committee on Antimicrobial Susceptibility Testing
IQR	Interquartile range
MALDI-TOF MS	Matrix-assisted laser desorption ionization time of flight mass spectrometry
MDR	Multidrug resistance
PDR	Pan-drug-resistant
µg	Microgram
XDR	Extensively drug-resistant
