# Supplementary material for: Antibiotic Resistance Profiles of Commensal and Pathogenic Bacteria Isolated from Wild Boar Carcasses in Campania Region, Southern Italy
Source: Antibiotics (Basel). 2026 Jan 7;15(1):65. doi: 10.3390/antibiotics15010065 (PMC12837608; doi:10.3390/antibiotics15010065)
Supplement: Supplementary file 1 [file antibiotics-15-00065-s001.zip › antibiotics-4039160-supplementary Table S1.pdf]

Resistance of different bacterial genera to different classes of antibiotics

Escherichia coli

Code	Antibiotic	Class	Class_2	Resistant	Percentage (tot =57)
AMC	Amoxicillin-clavulanate	Penicillin	Penicillin-Beta lactamase	38	66.67
AMI	Amikacin	Aminoglycoside	Aminoglycoside	18	31.58
AMP	Ampicillin	Penicillin	Penicillin	8	14.04
AZT	Aztreonam	Monobactam	Monobactam	31	54.39
CAZ	Ceftazidime	Cephalosporin	Cephalosporin_3rd	27	47.37
CFO	Cefoxitin	Cephalosporin	Cephalosporin_2nd	8	14.04
CIP	Ciprofloxacin	Quinolone	Quinolone	10	17.54
CTX	Cefotaxime	Cephalosporin	Cephalosporin_3rd	16	28.07
GEN	Gentamicin	Aminoglycoside	Aminoglycoside	19	33.33
IMI	Imipenem	Carbapenem	Carbapenem	0	0
LEV	Levofloxacin	Quinolone	Quinolone	20	35.09
MRP	Meropenem	Carbapenem	Carbapenem	0	0
PTZ	Piperacillin-Tazobactam	Penicillin	Penicillin-Beta lactamase	25	43.86
SXT	Trimethoprim-suphamethoxazole	Sulfonamide	Aminopyrimidine_sulphonamide	5	8.77
TIG	Tigecycline	Tetracycline	Tetracycline	6	10.53

Pantoea agglomerans

Code	Antibiotic	Class	Class_2	Resistant	Percentage (tot =10)
AMC	Amoxicillin-clavulanate	Penicillin	Penicillin-Beta lactamase	9	90
AMI	Amikacin	Aminoglycoside	Aminoglycoside	2	20
AMP	Ampicillin	Penicillin	Penicillin	3	30
AZT	Aztreonam	Monobactam	Monobactam	2	20
CAZ	Ceftazidime	Cephalosporin	Cephalosporin_3rd	6	60
CFO	Cefoxitin	Cephalosporin	Cephalosporin_2nd	2	20
CIP	Ciprofloxacin	Quinolone	Quinolone	4	40
CTX	Cefotaxime	Cephalosporin	Cephalosporin_3rd	1	10
GEN	Gentamicin	Aminoglycoside	Aminoglycoside	6	60
IMI	Imipenem	Carbapenem	Carbapenem	0	0
LEV	Levofloxacin	Quinolone	Quinolone	0	0
MRP	Meropenem	Carbapenem	Carbapenem	0	0
PTZ	Piperacillin-Tazobactam	Penicillin	Penicillin-Beta lactamase	5	50
SXT	Trimethoprim-suphamethoxazole	Sulfonamide	Aminopyrimidine_sulphonamide	0	0
TIG	Tigecycline	Tetracycline	Tetracycline	0	0

Enterobacter ludwigii

Code	Antibiotic	Class	Class_2	Resistant	Percentage (tot =1)
AMC	Amoxicillin-clavulanate	Penicillin	Penicillin-Beta lactamase	1	100

AMI	Amikacin	Aminoglycoside	Aminoglycoside	0	0
AMP	Ampicillin	Penicillin	Penicillin	1	100
AZT	Aztreonam	Monobactam	Monobactam	1	100
CAZ	Ceftazidime	Cephalosporin	Cephalosporin_3rd gen	1	100
CFO	Cefoxitin	Cephalosporin	Cephalosporin_2nd gen	1	100
CIP	Ciprofloxacin	Quinolone	Quinolone	0	0
CTX	Cefotaxime	Cephalosporin	Cephalosporin_3rd gen	1	100
GEN	Gentamicin	Aminoglycoside	Aminoglycoside	1	100
IMI	Imipenem	Carbapenem	Carbapenem	1	100
LEV	Levofloxacin	Quinolone	Quinolone	0	0
MRP	Meropenem	Carbapenem	Carbapenem	0	0
PTZ	Piperacillin-Tazobactam	Penicillin	Penicillin-Beta lactamase	1	100
SXT	Trimethoprim-suphamethoxazole	Sulfonamide	Aminopyrimidine-sulphonamide	0	0
TIG	Tigecycline	Tetracycline	Tetracycline	1	100

Staphylococcus spp. (several species)

Code	Antibiotic	Class	Class_2	Resistant	Percentage (tot =67)
AMI	Amikacin	Aminoglycoside	Aminoglycoside	5	7.46
ERY	Erythromycin	Macrolide	Macrolide	35	52.24
CFO	Cefoxitin	Cephalosporin	Cephalosporin_2nd gen	11	16.42
CIP	Ciprofloxacin	Quinolone	Quinolone	20	29.85
GEN	Gentamicin	Aminoglycoside	Aminoglycoside	11	16.42
LEV	Levofloxacin	Quinolone	Quinolone	23	34.33
LIZ	Linezolid	Oxazolidinones	Oxazolidinones	38	56.72
SXT	Trimethoprim-suphamethoxazole	Sulfonamide	Aminopyrimidine-sulphonamide	11	16.42
TIG	Tigecycline	Tetracycline	Tetracycline	20	29.85
TET	Tetracycline	Tetracycline	Tetracycline	29	43.28

Rothia nasimurium

Code	Antibiotic	Class	Class_2	Resistant	Percentage (tot =2)
AMI	Amikacin	Aminoglycoside	Aminoglycoside	0	0
ERY	Erythromycin	Macrolide	Macrolide	1	50
CFO	Cefoxitin	Cephalosporin	Cephalosporin_2nd	1	50
CIP	Ciprofloxacin	Quinolone	Quinolone	1	50
GEN	Gentamicin	Aminoglycoside	Aminoglycoside	1	50
LEV	Levofloxacin	Quinolone	Quinolone	1	50
LIZ	Linezolid	Oxazolidinones	Oxazolidinones	1	50
SXT	Trimethoprim-suphamethoxazole	Sulfonamide	Aminopyrimidine_sulphonamide	0	0
TIG	Tigecycline	Tetracycline	Tetracycline	1	50
TET	Tetracycline	Tetracycline	Tetracycline	1	50

Macrococcus canis

Code	Antibiotic	Class	Class_2	Resistant	Percentage (tot =2)
AMI	Amikacin	Aminoglycoside	Aminoglycoside	0	0
ERY	Erythromycin	Macrolide	Macrolide	2	100
CFO	Cefoxitin	Cephalosporin	Cephalosporin_2nd	2	100
CIP	Ciprofloxacin	Quinolone	Quinolone	0	0
GEN	Gentamicin	Aminoglycoside	Aminoglycoside	1	50
LEV	Levofloxacin	Quinolone	Quinolone	1	50
LIZ	Linezolid	Oxazolidinones	Oxazolidinones	0	0
SXT	Trimethoprim-suphamethoxazole	Sulfonamide	Aminopyrimidine_sulphonamide	0	0
TIG	Tigecycline	Tetracycline	Tetracycline	1	50
TET	Tetracycline	Tetracycline	Tetracycline	0	0

Kocuria rhizophila

Code	Antibiotic	Class	Class_2	Resistant	Percentage (tot =4)
AMI	Amikacin	Aminoglycoside	Aminoglycoside	0	0
ERY	Erythromycin	Macrolide	Macrolide	4	100
CFO	Cefoxitin	Cephalosporin	Cephalosporin_2nd	3	75
CIP	Ciprofloxacin	Quinolone	Quinolone	4	100
GEN	Gentamicin	Aminoglycoside	Aminoglycoside	4	100
LEV	Levofloxacin	Quinolone	Quinolone	4	100
LIZ	Linezolid	Oxazolidinones	Oxazolidinones	4	100
SXT	Trimethoprim-suphamethoxazole	Sulfonamide	Aminopyrimidine_sulphonamide	3	75
TIG	Tigecycline	Tetracycline	Tetracycline	1	25
TET	Tetracycline	Tetracycline	Tetracycline	3	75

Arthrobacter koreensis

Code	Antibiotic	Class	Class_2	Resistant	Percentage (tot =1)
AMI	Amikacin	Aminoglycoside	Aminoglycoside	0	0
ERY	Erythromycin	Macrolide	Macrolide	1	100
CFO	Cefoxitin	Cephalosporin	Cephalosporin_2nd gen	1	100
CIP	Ciprofloxacin	Quinolone	Quinolone	1	100
GEN	Gentamicin	Aminoglycoside	Aminoglycoside	1	100
LEV	Levofloxacin	Quinolone	Quinolone	1	100
LIZ	Linezolid	Oxazolidinones	Oxazolidinones	1	100
SXT	Trimethoprim-suphamethoxazole	Sulfonamide	Aminopyrimidine-sulphonamide	1	100
TIG	Tigecycline	Tetracycline	Tetracycline	0	0
TET	Tetracycline	Tetracycline	Tetracycline	0	0

Paenarthrobacter ilicis

Code	Antibiotic	Class	Class_2	Resistant	Percentage (tot =1)
AMI	Amikacin	Aminoglycoside	Aminoglycoside	0	0
ERY	Erythromycin	Macrolide	Macrolide	1	100
CFO	Cefoxitin	Cephalosporin	Cephalosporin_2nd	1	100
CIP	Ciprofloxacin	Quinolone	Quinolone	1	100
GEN	Gentamicin	Aminoglycoside	Aminoglycoside	0	0
LEV	Levofloxacin	Quinolone	Quinolone	0	0
LIZ	Linezolid	Oxazolidinones	Oxazolidinones	1	100
SXT	Trimethoprim-suphamethoxazole	Sulfonamide	Aminopyrimidine_sulphonamide	0	0
TIG	Tigecycline	Tetracycline	Tetracycline	1	100
TET	Tetracycline	Tetracycline	Tetracycline	1	100

Pseudomonas **spp. (several species)**

Code	Antibiotic	Class	Class_2	Resistant	Percentage (tot =7)
AMI	Amikacin	Aminoglycoside	Aminoglycoside	0	0
AZT	Aztreonam	Monobactam	Monobactam	4	57.14
CAZ	Ceftazidime	Cephalosporin	Cephalosporin_3rd	4	57.14
CIP	Ciprofloxacin	Quinolon	Quinolon	2	28.57
IMI	Imipenem	Carbapenem	Carbapenem	1	14.29
LEV	Levofloxacin	Quinolon	Quinolon	1	14.29
MRP	Meropenem	Carbapenem	Carbapenem	0	0
PTZ	Piperacillin-Tazobactam	Penicillin	Penicillin-Beta lactamase	3	42.86

Alcaligenes faecalis

Code	Antibiotic	Class	Class_2	Resistant	Percentage (tot =20)
AMI	Amikacin	Aminoglycoside	Aminoglycoside	1	5
AZT	Aztreonam	Monobactam	Monobactam	13	65
CAZ	Ceftazidime	Cephalosporin	Cephalosporin_3rd	13	65
CIP	Ciprofloxacin	Quinolone	Quinolone	18	90
IMI	Imipenem	Carbapenem	Carbapenem	0	0
LEV	Levofloxacin	Quinolone	Quinolone	4	20
MRP	Meropenem	Carbapenem	Carbapenem	0	0
PTZ	Piperacillin-Tazobactam	Penicillin	Penicillin-Beta lactamase	15	75

Bacillus **spp. (several species)**

Code	Antibiotic	Class	Class_2	Resistant	Percentage (tot =14)
ERY	Erythromycin	Macrolide	Macrolide	6	42.86
CIP	Ciprofloxacin	Quinolone	Quinolone	5	35.71

IMI	Imipenem	Carbapenem	Carbapenem	6	42.86
LEV	Levofloxacin	Quinolone	Quinolone	6	42.86
LIZ	Linezolid	Oxazolidinone	Oxazolidinone	6	42.86
MRP	Meropenem	Carbapenem	Carbapenem	2	14.29
VAN	Vancomycin	Glycopeptide	Glycopeptide	0	0

Exiguobacterium mexicanum

Code	Antibiotic	Class	Class_2	Resistant	Percentage (tot=6)
ERY	Erythromycin	Macrolide	Macrolide	4	66.67
CIP	Ciprofloxacin	Quinolone	Quinolone	3	50
IMI	Imipenem	Carbapenem	Carbapenem	0	0
LEV	Levofloxacin	Quinolone	Quinolone	3	50
LIZ	Linezolid	Oxazolidinone	Oxazolidinone	3	50
MRP	Meropenem	Carbapenem	Carbapenem	0	0
VAN	Vancomycin	Glycopeptide	Glycopeptide	0	0

Streptococcus spp. (several species)

Code	Antibiotic	Class	Class_2	Resistant	Percentage (tot=3)
BZPEN	Benzyl penicillin	Penicillin	Penicillin	1	33.33
ERY	Erythromycin	Macrolide	Macrolide	0	0
LEV	Levofloxacin	Quinolone	Quinolone	1	33.33
LIZ	Linezolid	Oxazolidinone	Oxazolidinone	1	33.33
SXT	Trimethoprim-suphamethoxazole	Sulfonamide	Aminopyrimidine-sulphonamide	1	33.33
TET	Tetracycline	Tetracycline	Tetracycline	1	33.33
TIG	Tetracycline	Tetracycline	Tetracycline	1	33.33
VAN	Vancomycin	Glycopeptide	Glycopeptide	0	0

Enterococcus spp. (Multiple species)

Code	Antibiotic	Class	Class_2	Resistant	Percentage (tot=5)
AMP	Ampicillin	Penicillin	Penicillin	0	0
CIP	Ciprofloxacin	Quinolone	Quinolone	1	20
GEN	Gentamycin	Aminoglycoside	Aminoglycoside	0	0
IMI	Imipenem	Carbapenem	Carbapenem	1	20
LEV	Levofloxacin	Quinolone	Quinolone	0	0
LIZ	Linezolid	Oxazolidinone	Oxazolidinone	3	60
TIG	Tigecycline	Tetracycline	Tetracycline	3	60
VAN	Vancomycin	Glycopeptide	Glycopeptide	3	60
